# Supplementary material for: ZP4 confers structural properties to the zona pellucida essential for embryo development
Source: eLife. 2019 Oct 22;8:e48904. doi: 10.7554/eLife.48904 (PMC6805156; doi:10.7554/eLife.48904)
Supplement: Transparent reporting form [file elife-48904-transrepform.pdf]

eLIFE
1st Floor
24 Hills Road
Cambridge CB2 1JP, UK
P 01223 855340
W elifesciences.org
T @elife

eLife's transparent reporting form

We encourage authors to provide detailed information *within their submission* to facilitate the interpretation and replication of experiments. Authors can upload supporting documentation to indicate the use of appropriate reporting guidelines for health-related research (see EQUATOR Network), life science research (see the BioSharing Information Resource), or the ARRIVE guidelines for reporting work involving animal research. Where applicable, authors should refer to any relevant reporting standards documents in this form.

If you have any questions, please consult our Journal Policies and/or contact us: editorial@elifesciences.org.

Sample-size estimation

- You should state whether an appropriate sample size was computed when the study was being designed
- You should state the statistical method of sample size computation and any required assumptions
- If no explicit power analysis was used, you should describe how you decided what sample (replicate) size (number) to use

Please outline where this information can be found within the submission (e.g., sections or figure legends), or explain why this information doesn't apply to your submission:

Sample size was not estimated before-hand because the phenotypic outcome of the intervention (ZP4 KO) was completely unknown, so we could not estimate an expected variation of the parameters analyzed (related to fertility) to determine a sample size. Given that most fertility parameters analyzed implied animal killing, we kept sample sizes at the minimum (3-5 animals analyzed per group) to achieve statistical and biological significance.

Replicates

- You should report how often each experiment was performed
- You should include a definition of biological versus technical replication
- The data obtained should be provided and sufficient information should be provided to indicate the number of independent biological and/or technical replicates
- If you encountered any outliers, you should describe how these were handled
- Criteria for exclusion/inclusion of data should be clearly stated
- High-throughput sequence data should be uploaded before submission, with a private link for reviewers provided (these are available from both GEO and ArrayExpress)

Please outline where this information can be found within the submission (e.g., sections or figure legends), or explain why this information doesn't apply to your submission:

The number of biological replicates are indicated below. All data was included (none was excluded). Given the type of parameter analyzed (fertility) technical replicates do not apply in most cases (i.e., there is one measure per biological replicate).

Gene ablation confirmation: Immunohistochemistry in 3 biological replicates (3 ovaries from different animals, line 291, Fig. 1B) and proteomics analysis in 1 technical replicate (line 307, table supplement 1).
Pregnancy (pups delivered): 10 animals (biological replicates)/group. Line 110, Fig. 2A.
Ovulation, embryonic cleavage and development to blastocysts: 5 biological replicates (5 animals/group). Lines 112 & 320. Fig. 2B-D.
Follicular counts: 4 animals (biological replicates)/group, 4 section per ovary. Lines 326-327, Fig. 2E-F.
Pronuclei formation: 3 animals (biological replicates)/group. Lines 127 & 327, Fig. 2G.
In vivo development to Day 6 and Day 6 embryo size: 4 animals (biological replicates)/group. Lines 138-139 & 331. Fig. 3A-B.
ZP thickness: 30 embryos (biological replicates)/group. Line 337
Electron microscopy: 5 embryos (biological replicates)/group. Line 339.
ZP permeability test: 3 technical replicates of 3 embryos per group (9 biological replicates/group). Line 344.

Statistical reporting

- Statistical analysis methods should be described and justified
- Raw data should be presented in figures whenever informative to do so (typically when N per group is less than 10)
- For each experiment, you should identify the statistical tests used, exact values of N, definitions of center, methods of multiple test correction, and dispersion and precision measures (e.g., mean, median, SD, SEM, confidence intervals; and, for the major substantive results, a measure of effect size (e.g., Pearson's r, Cohen's d)
- Report exact p-values wherever possible alongside the summary statistics and 95% confidence intervals. These should be reported for all key questions and not only when the p-value is less than 0.05.

Please outline where this information can be found within the submission (e.g., sections or figure legends), or explain why this information doesn't apply to your submission:

One-way ANOVA following Tukey´s post hoc test or t-test were used for all analyses to compare 3 or 2 groups, respectively, except for permeability test (binary variable), where Chi-square test were used. Differences were considered statistically significant at p>0.05. Lines 365-367 and Figure legends.

(For large datasets, or papers with a very large number of statistical tests, you may upload a single table file with tests, Ns, etc., with reference to sections in the manuscript.)

Group allocation

- Indicate how samples were allocated into experimental groups (in the case of clinical studies, please specify allocation to treatment method); if randomization was used, please also state if restricted randomization was applied
- Indicate if masking was used during group allocation, data collection and/or data analysis

Please outline where this information can be found within the submission (e.g., sections or figure legends), or explain why this information doesn't apply to your submission:

Samples (oocytes or embryos enclosed by WT, Hz or KO ZP) were allocated to each experimental group (WT, Hz or KO) based on genotyping of the donor females.

Additional data files ("source data")

- We encourage you to upload relevant additional data files, such as numerical data that are represented as a graph in a figure, or as a summary table
- Where provided, these should be in the most useful format, and they can be uploaded as "Source data" files linked to a main figure or table
- Include model definition files including the full list of parameters used
- Include code used for data analysis (e.g., R, MatLab)
- Avoid stating that data files are "available upon request"

Please indicate the figures or tables for which source data files have been provided:

We are providing "source data" for protein identification (Table supplement 1) and genomic analysis (Table supplement 2) as Excel files.

eLife Sciences Publications, Ltd is a limited liability non-profit non-stock corporation incorporated in the State of Delaware, USA, with company number 5030732, and is registered in the UK with company number FC030576 and branch number BR015634 at the address 1st Floor, 24 Hills Road, Cambridge CB2 1JP | August 2014
